# Mariage consanguin et morbi-mortalité, courte revue de la littérature à partir d'une association exceptionnelle: syndrome de Usher et Neurofibromatose de Von Recklinghausen

**DOI:** 10.11604/pamj.2016.23.99.9025

**Published:** 2016-03-15

**Authors:** Pépin-Williams Atipo-Tsiba

**Affiliations:** 1 Service d'Ophtalmologie, CHU de Brazzaville, Congo

**Keywords:** Consanguinité, maladies génétiques rares, syndrome de Usher, Inbreeding, rare genetic diseases, Usher's syndrome

## Abstract

Le syndrome de Usher est défini par l'association d'une surdité de perception congénitale de sévérité variable évolutive ou non et d'une rétinopathie pigmentaire progressivement cécitante. La Neurofibromatose de Von Recklinghausen ou Neurofibromatose de type I est la principale forme clinique des neurofibromatoses avec environ 90% des cas. Ces deux maladies sont d'origine génétique avec des prévalences très basses. La probabilité pour qu'un seul et même individu souffre à la fois de ces maladies est exceptionnelle. Comme toutes les maladies génétiques, la consanguinité augmente de façon assez sensible la probabilité de leur apparition. Le mariage consanguin est encore largement répondu au Maghreb et dans certaines régions d'Afrique de l'Ouest. Cette observation rapporte un cas exceptionnel de cette association chez un homme de 40 ans originaire de la Mauritanie né d'une union consanguine.

## Abstract (en)

Usher syndrome is defined by the association of a progressive or non-progressive congenital sensorineural hearing loss with variable severity and a gradually blinding pigmentary retinopathy. Von Recklinghausen neurofibromatosis or Neurofibromatosis type 1 is the major clinically form of neurofibromatosis which occurs in approximately 90% of cases. Both types of disease are genetic in origin with very low prevalence. The probability of co-occurrence of these diseases in a single individual is exceptional. Inbreeding, as well as all genetic diseases, increases quite significantly the probability of their occurrence. Consanguineous marriages are still widespread in Maghreb and in some regions of the western African. This observation reports an exceptional case of this association in a 40-year-old man of Mauritanian origin born from a consanguineous union.

## Introduction

Le syndrome d'Usher (SU) est défini par l'association d'une surdité de perception congénitale de sévérité variable évolutive ou non et d'une rétinopathie pigmentaire progressivement cécitante [1]. Il en existe trois types cliniques selon l'atteinte vestibulaire et le degré de surdité. Le type II qui nous intéresse dans cet article est contrôlé par un gène au locus D1S81 du bras long du chromosome 1. Dans cette forme, à la différence des deux autres, l'acquisition du langage est possible et la rétinopathie pigmentaire apparait plus tardivement elle est progressive et devient gênante vers l’âge de 30 ans [2, 3]. La neurofibromatose est une maladie génétique autosomique dominant [4]. Il en existe deux types. La Neurofibromatose de Von Recklinghausen (NVR) décrit dans ce travail est le type I. Elle représente environ 90% des formes cliniques et est due à une anomalie au niveau du chromosome 17. Ses atteintes oculaires les plus fréquents sont représentées par les nodules iriens de Lisch, le névrome plexiforme de la paupière supérieure, le gliome des voies optiques. La coexistence de ces deux maladies chez un même individu est exceptionnelle, nous n'avons trouvé aucun cas dans la littérature. Cette observation rapporte une association exceptionnelle SU-NVR chez un homme âgé de 40 ans originaire de la Mauritanie né d'une union consanguine.

## Patient et observation

**Figure 1:**
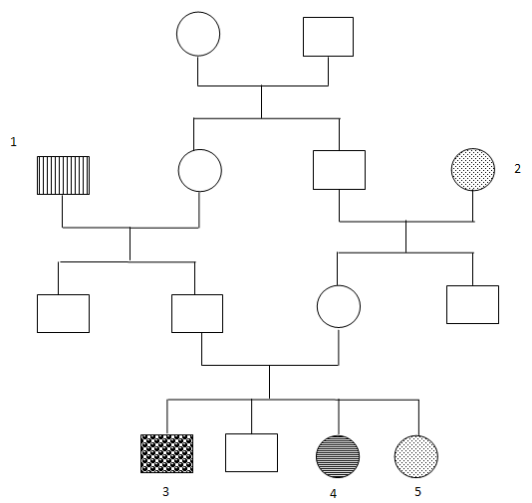
Arbre généalogique de la famille de Monsieur M

Mr. M âgé de 40 ans était admis notre service pour une cécité. Cette cécité avait débuté vers l’âge de 21 ans. Elle était bilatérale, rapidement progressive, indolore, sans fièvre et sans atteinte de l’état général. Elle était associée à une surdité, rapidement handicapante, contraignant son entourage à parler très fort pour sa compréhension. Mr M est né d'une fratrie de 4 enfants, issus d'un mariage consanguin. Son grand-père paternel souffre d'une neurofibromatose de Von Recklinghausen, et sa grand-mère maternelle souffre d′une rétinite pigmentaire. Une sœur de monsieur M est albinos, et une autre souffre d'une rétinite pigmentaire. Figure 1 représente l′arbre généalogique de Monsieur M. Le status clinique de ce patient était le suivant (des deux côtés): une absence de perception lumineuse, une rétinite pigmentaire, une surdité de perception moyenne (perte de 50 décibels) sans atteinte vestibulaire. Les neurofibromes recouvraient toute sa surface corporelle.

## Discussion

Le syndrome d'Usher a été décrit pour la première fois en 1858 par Von Graffe [1]. Usher est le premier à appréhender le caractère héréditaire de cette association pathologique et le décrit comme un syndrome spécifique [5]. Cette maladie est à l'origine de 3 à 6% des surdités congénitales, et représente environ 50% de la population sourde-aveugle aux USA [3]. Sa prévalence (P1) est de 1 naissance/1000 [2]. Von Recklinghausen fut le premier en 1882 à décrire la neurofibromatose qui finira par porter son nom [6]. La Neurofibromatose de Von Recklinghausen décrit dans ce travail est le type I, sa prévalence (P2) est de 1 naissance/4>950 [4]. Si l'on considérait un modèle de mathématique simple, le “théorème d'indépendance en probabilité” (Accessed 31/01/2016: www.medespace.fr/../probabilité-conditionnelle-indépendance ), la probabilité (P) pour qu'un seul et même individu souffre de ces deux maladies peut être calculée par la formule suivante: P (P1UP2) = P1 X P2 soit 1 naissance/4.950.000 (une naissance sur près de cinq millions). Cette probabilité est extrêmement faible. La consanguinité a probablement joué un rôle déterminant dans l’éclosion de cette association [7]. En Grande-Bretagne près de 35% des enfants atteints de maladies génétiques récessives rares sont issus d'un mariage consanguin (Accessed 31/01/2016: http://www.bivouac-id.com/mariages-consanguins-et-tares-genetiques/). Au Maroc les maladies liées à la consanguinité occupent une place importante dans le système de santé. La plus terrible d'entre elles est la myopathie de Duchenne, les enfants en meurent vers 15 ans (Accessed 31/01/2016: http://www.maghress.com/fr/leconomiste/34435). Environ 50% des mariages en Mauritanie sont consanguins (Accessed 31/01/2016: http://www.bivouac-id.com/mariages-consanguins-et-tares-genetiques/). Certains facteurs pourraient expliquer la consanguinité, la structure familiale traditionnelle, l′isolement géographique, le contexte économique les pauvres se marient entre eux et les riches font de même, et enfin l′isolement religieux. Il aurait été intéressant de déterminer le gene à l′origine de cette association, Monsieur M a refusé tout prélèvement sanguin au motif qu′aucun traitement curatif ne lui serait proposé par la suite.

## Conclusion

Les mariages consanguins sont parfois la source de maladie génétique rare et le plus souvent grave. Les médecins, les leaders politiques et religieux devraient unir les efforts afin d'interdire ces unions entre les membres d'une même famille.
